# Mentoring Young African American Men and Transgender Women Who Have Sex With Men on Sexual Health: Formative Research for an HIV Mobile Health Intervention for Mentors

**DOI:** 10.2196/17317

**Published:** 2020-12-17

**Authors:** Michelle R Kaufman, Albert Casella, John Mark Wiginton, Wenjian Xu, David L DuBois, Renata Arrington-Sanders, Jeannette Simon, Deb Levine

**Affiliations:** 1 Department of Health, Behavior & Society Johns Hopkins Bloomberg School of Public Health Baltimore, MD United States; 2 Department of Sociology & Psychology School of Public Administration Sichuan University Chengdu China; 3 Division of Community Health Sciences and Institute for Health Research and Policy University of Illinois Chicago Chicago, IL United States; 4 School of Medicine Johns Hopkins University Baltimore, MD United States; 5 Ananizach, LLC Baltimore, MD United States; 6 DKF Consulting Oakland, CA United States

**Keywords:** mentoring, HIV, mobile app, mHealth, men who have sex with men, transgender, African Americans

## Abstract

**Background:**

African American men who have sex with men (MSM) and transgender women bear a disproportionate burden of HIV. Young MSM account for 75% of this burden for youth. When youths lack socially protective resources such as strong networks of adults, including parents, teachers, or community members, mentors may play a critical role in promoting health behaviors. This is especially true for youth at risk for HIV, such as African American youth with sexual and gender minority (SGM) identities. In the past decade, natural mentoring and mentoring programs have proliferated as a key prevention and intervention strategy to improve outcomes for young people at risk for poor academic, social, and health issues. Mentors appear to be able to facilitate health promotion among young SGM by modeling healthy behaviors; however, mentors’ knowledge and resource needs regarding sexual health topics including HIV are understudied, as is the potential role of mobile technology in enhancing mentoring relationships and the ability of mentors to learn about sensitive issues faced by youth.

**Objective:**

The aim of this study is to explore how mentoring plays a role in the sexual health of African American SGM youth and understand how mentoring relationships can be strengthened through mobile technology to promote youth HIV prevention behaviors.

**Methods:**

In-depth interviews were conducted with African American SGM youth mentees (n=17) and mentors (n=20) to such youths in 3 Mid-Atlantic cities. Mentee interviews focused on discussions regarding sexual health and HIV and how a mentor could broach such topics. Mentor interviews explored whether sexual health and HIV are currently mentoring topics, mentors’ knowledge and confidence in mentoring on these issues, and barriers to discussions. All participants were asked if a mobile app could help facilitate mentoring on sensitive health issues, particularly HIV and sexual health. Data were transcribed, coded, and analyzed for relevant themes.

**Results:**

Sexual health was a common topic in mentoring relationships, occurring more in natural mentorships than in mentoring program pairs. Mentors and mentees felt positive about such discussions. Mentors expressed having limited knowledge beyond condom use and HIV testing, and expressed a need for more complete resources. Both mentors and mentees had mixed comfort levels when discussing sexual health. Sufficient trust and shared lived experiences made discussions easier. Mentees have multifaceted needs; however, mentors stated that an app resource that provided self-training, resources, support from other mentors, and tips for better mentoring could prove beneficial.

**Conclusions:**

For the African American SGM community, access to natural mentors is crucial for young people to learn healthy behaviors. A mobile resource to assist mentors in confidently having discussions with mentees may be a promising way to promote healthy practices.

## Introduction

### Background

Young African American gay, bisexual, and other men who have sex with men (MSM) continue to bear a disproportionate burden of HIV [1-5]. In 2018, 26% of all incident HIV infections were among African American MSM, accounting for 37% of incident HIV infections among all MSM. In addition, 75% of African American MSM diagnosed as having HIV were younger than 35 years [5]. Between 2000 and 2010, there was a 133% increase in HIV infections among young African American men aged between 13 to 24 years. This number has since seen a downward trend (11%); however, these young men continue to bear a significant burden of infection in the United States [6,7]. Although less is known about HIV incidence among African American transgender women, a recent meta-analysis found a 44% HIV prevalence in this population, compared with 7% among White transgender women [8]. Moreover, research has highlighted increased vulnerabilities for HIV acquisition among young transgender women, including those reporting African American race or ethnicity [9].

These sexual health disparities are contextualized by oppressive sociostructural factors with which young, urban African American MSM and transgender women must contend, including racism, homophobia, transphobia, and classism [10,11]. These factors underpin experiences of stigma, bullying, rejection, abuse, and home eviction [12-14]; a lack of appropriately tailored sexuality education and accessible, culturally competent health care [13-15]; and fractured support networks, socioeconomic challenges, and limited community resources [16,17]. These conditions set the stage for poor sexual and other health outcomes for young African American MSM and transgender women, including a greater likelihood of engaging in sex without a condom, exchanging sex for money and basic needs, or an increased number of risky encounters with multiple sex partners, all of which contribute to disparities in HIV infection [12,18,19].

Mentoring could be leveraged to mitigate the effects of such conditions and facilitate the sexual health of these populations and other sexual and gender minority (SGM) youth. In the youth development context, a *mentor* is commonly defined as a nonparental adult who builds a relationship with a young person, plays a supportive role in their life, and provides guidance and encouragement to cultivate positive and healthy development [20]. In the past decade, mentoring in formal programs and naturally occurring relationships between youth and trusted adults has proliferated as a key prevention and intervention strategy for young people at risk of poor academic, social, and health outcomes [20]. A meta-analysis by DuBois et al [20] synthesized evaluation findings of one-to-one and group-based youth mentoring programs and found positive, though modest, effects on emotional, psychological, behavioral, social, academic, and career outcomes across diverse mentoring program types. Mentors of SGM youth could likewise provide social and emotional support, sex and sexuality-related guidance and education, assistance with accessing health care and other resources, and social capital through connection to a broader social network. In fact, SGM young adults with a mentor report achieving higher levels of education than those without mentors, with the strongest difference observed among young MSM compared with their heterosexual peers [21]. Mentorship has also been associated with positive sexual identity development and improved quality of life among MSM [22].

Few mentoring programs tailored to the needs of SGM youth exist today, despite the calls for such programs over the past two decades [23-25]. Furthermore, extant non–SGM-specific formal mentoring programs may be perceived as unsafe by SGM youth, particularly those who are also racial or ethnic minorities [26]. This lack of mentoring resources may explain why available data suggest that 60% to 80% of SGM youth tend to receive mentorship from individuals with whom they have naturally occurring relationships, such as nonparental relatives, teachers, and individuals in the lesbian, gay, bisexual, transgender, and queer (LGBTQ) community [21,27,28].

Young African American MSM have explicitly expressed the need for mentors because of family rejection and consequent lack of guidance [25], and they have articulated a need for support and direction regarding sex, sexuality, and HIV prevention [29]. As in other SGM youth populations, some young African American MSM have found mentors naturally [30]. For others, mentorship has come through membership in constructed families (families formed with nonbiologically related individuals) or participation in the house and ball community (an LGBTQ underground subculture) [31,32]. Individuals receiving mentorship in these latter contexts have indicated that such support has helped them build healthy relationships, engage in HIV-preventive behaviors, and has conferred social, emotional, and instrumental support [32-34]. This limited body of research leaves much unknown about mentoring relationships among young African American MSM, and there is a general lack of research on mentoring relationships among young African American transgender women.

Previous research has shown the perceived benefits of mobile phone–based health interventions, and such interventions have great potential for improving young people’s sexual health outcomes [35-38]. Although the incorporation of technology into youth mentoring relationships is new [39], everyday life is now intimately intertwined with mobile technology. The intersection between youth mentoring, mobile technology, and improvements in sexual health deserves further exploration.

### Study Objectives

This research sought to explore the role of mentoring among African American MSM and transgender female populations. In addition, this research aimed to learn how mentoring on sexual health issues among this group takes place, if at all, and how mobile technology might enhance mentoring on such sensitive issues. The objectives of this study are to (1) explore the extent to which existing mentoring relationships play a role in the sexual health of African American SGM youth who have sex with men and (2) understand ways in which these mentoring relationships can be strengthened to promote HIV prevention behaviors among the youth, particularly through the use of mobile technology resources. Findings from this formative research informed the development of a mobile app designed to facilitate sexual health conversations between African American SGM and their mentors.

## Methods

### Participant Description

Participants were mentors and mentees living in Baltimore, Philadelphia, and Washington, DC. Mentee participants were included if they identified as African American; identified as cisgender male, transgender female, or nonbinary assigned male at birth; were aged between 15-24 years; expressed a sexual interest in men; and reported currently having a nonfamilial adult mentor whom they had known for at least three months. Mentor participants were included if they were aged 18 years or older, serving as a nonfamilial mentor to a youth meeting the mentee inclusion criteria, and had at least three months of mentoring experience with such a mentee at the time of participation. The sexual and gender identities of mentors were not considered for eligibility. Study participation was not contingent on one’s mentor or mentee also participating.

### Recruitment

Participants were recruited through organizations focused on mentoring and/or LGBTQ services in each city and through passive recruitment methods (eg, posted flyers, flyer distribution at LGBTQ events, advertisements on streaming radio, and social media). Participants in natural mentoring relationships were screened to determine whether the relationship met the definition of mentoring (ie, a relationship between the youth and a nonfamilial adult that focuses on the positive development and well-being of the youth). Formal mentoring program participants were not screened for mentoring relationships because of programs completing this process as part of onboarding procedures.

### Data Collection Procedures

Participants completed an oral consent process, followed by an individual, semistructured qualitative interview lasting 45-60 min. The interviews began with a small number of structured questions to assess the characteristics of the mentors and mentees and their relationships. Interviewers were an African American gay male (for the mentee interviews) and an African American cisgender female with more than 25 years of mentoring program experience (for the mentor interviews). Mentee interviews focused on discussions with their mentors regarding sexual health and HIV risk and/or treatment (or why such conversations were absent) and how a mentor could broach such conversations in a youth-friendly manner. Mentor interviews explored whether sexual health and HIV risk and/or treatment are mentoring topics, how much mentors know about these topics, their confidence in mentoring on these issues, and current and/or potential barriers to such discussions. Consistent with the intervention development aim of the research, all participants were also asked whether and how a mobile app might facilitate mentoring on sexual health topics. An abridged interview guide is available in Multimedia Appendix 1.

### Data Analysis Procedures

All interviews were audio recorded, transcribed verbatim, and imported into Atlas.ti (version 8.2). Data analysis included inductive and deductive coding techniques, whereby team members independently read transcripts and reached a consensus on the code list. Codebook development was based on the aims of this study and previous literature. Two research team members (both males, 1 gay and 1 heterosexual) independently coded the transcripts. To ensure reliability, 25% of the transcripts were randomly selected for double coding. Coding was considered reliable once the intercoder agreement reached ≥85%. Research team members reviewed code outputs and discussed their relevance to the objectives of this study. Additional data-driven codes were added throughout the analysis, provided there was agreement on their inclusion (in total, 88 codes were established). Data were analyzed thematically (each theme described under a subheading below), a method shown to allow for various epistemological and ontological stances [40].

## Results

### Participants

Mentee participants’ (n=17) mean age was 20.5 years (SD 1.85; range 18-24), and the mean age for mentors (n=20) was 30.1 years (SD 6.16; range 22-45). Recruiters did not identify any prospective participants in the age range of 15 to 17 years.

Participants were categorized into 4 types: formal mentor, naturally occurring mentor, formal mentee, and naturally occurring mentee. A description of the mentee and mentor demographics by city is available in Table 1. Only 1 mentee reported having a formal mentor, whereas 25% (5/20) of mentors reported being formal mentors. The remaining participants described their mentoring relationship as originating in a natural setting rather than through a formal mentoring program. Examples of these settings include the house-ball community, high school environments where the mentor was a teacher or coach, LGBTQ pride events, and the mentee’s social network. Nearly all participants reported being in their mentoring relationship for at least 1 year (15/17, 88% of mentees and 18/20, 90% of mentors), with an average duration of 2.5 years (mentee mean 2.34 years, SD 1.78; mentor mean 2.7 years, SD 2.08).

**Table 1:** Demographic and mentoring characteristics (N=37).

Characteristics		Mentee participants, n (%)	Mentor participants, n (%)
**City**			
	Baltimore	5 (29)	2 (10)
	Philadelphia	4 (24)	9 (45)
	Washington, DC	8 (47)	9 (45)
**Gender identity**			
	Cisgender male	8 (47)	14 (70)
	Transgender female	6 (35)	5 (25)
	Nonbinary	3 (18)	1 (5)
**Age (years)**			
	15-19	6 (35)	0 (0)
	20-29	11 (65)^a^	12 (60)
	30-39	N/A^b^	6 (30)
	40-49	N/A	2 (10)
**Educational attainment**			
	Less than high school	2 (12)	0 (0)
	High school graduate	14 (82)	8 (40)
	Some college	1 (6)	5 (25)
	Bachelor’s degree	0 (0)	5 (25)
	Master’s degree	0 (0)	2 (10)
**Mentorship type**			
	Formal	1 (6)	5 (25)
	Naturally occurring	16 (94)	15 (75)
**Length of relationship**			
	Less than 1 year	2 (12)	2 (10)
	1-2 years	10 (59)	10 (50)
	3-5 years	3 (18)	4 (20)
	5 or more years	2 (12)	4 (20)

^a^Mentee eligibility included a maximum age of 24 years.

^b^N/A: not applicable.

The reported communication frequency between mentors and mentees ranged from once per week to multiple times per day. Reported communication tended to be more frequent and used multiple modes (in person, text, and video chat) in naturally occurring relationships as compared with formal mentoring relationships.

We did not conduct a comparison of findings between the transgender and MSM participants given the limited sample (n=5) of transgender women. However, it should be noted that conversations with transgender mentees and their mentors tended to reveal a heightened focus on immediate basic needs.

### Sexual Health as a Common Topic of Conversation

Sexual health was often reported to be a prominent conversation topic between mentors and mentees. Almost all participants reported having conversations focused on sexual health within their mentoring relationship. These conversations seemed to be more common in longer (≥1 year), naturally occurring relationships, with a vast majority of longer mentorships reporting this discussion topic. All mentors who were part of a formal program cited the program’s structure (eg, curriculum) as an impediment to such conversations occurring, as the programs were focused on addressing other youth development issues (eg, academic performance). In line with this trend, only 1 participant from a formal mentoring relationship reported having such conversations. Although conversations on sexual health were reported by most participants, mentors revealed reluctance to broach these topics unsolicited:

I don’t want her to be like, ‘Why are you asking me questions [about HIV]?’...I’m comfortable with [having a conversation about sexual health], but I don’t want her to look like ‘why are you asking me that?’

Mentor, age 29 years, Washington, DC

Despite this reluctance, discussions of sexual health were reported to be initiated by mentors in the majority of cases. Mentees described these conversations as occurring in instances where the mentor and the mentee were talking about a mentee’s romantic relationship or recent sexual encounter. Mentors reported that they often initiated these conversations by embedding sexual health topics into a broader discussion of healthy relationships, such as the importance of open communication with partners and practicing safe sex.

There was little difference in conversation occurrence as reported by mentors and mentees. Mentors tended to describe these discussions as responses to observing risk factors in their mentee’s behavior, such as engaging with multiple partners. Mentees tended to describe discussions of sexual health as embedded in broader topics focused on dating, relationships, or current events.

### Positive Attitudes but Limited Mentor Knowledge Regarding Sexual Health

Attitudes toward conversations about sexual health topics between mentors and mentees, once broached, were overwhelmingly positive. Mentees cited confidence in the accuracy of information provided by their mentor because their mentor is a trusted adult with a shared life experience and has, in many cases, *been there*. Mentor participants typically expressed that they had a general knowledge of sexual health; however, a subset described themselves as less knowledgeable about sexual health care or standard prevention practice information. Almost all mentors mentioned that these conversations did not go into great detail or lead to service referral (eg, guiding their mentee to an HIV testing site).

Observing risk behavior in a mentee was a motivating factor for many mentors to introduce a conversation about sexual health. Key elements mentors used when planning to discuss sexual health were ensuring requisite trust was built in the relationship and a concern for the appearing objective:

You know, what’s really challenging is when you can see them [the mentee engaging in sexual risk behavior] without the other person knowing you can see, but you still have to remain neutral. I feel like [Mentee] and I have a trusting relationship, so I don’t think I would be afraid to bring it up. If we didn’t have that trust, it would be a different story.

Mentor, age 45 years, Washington, DC

I’ve talked to him multiple, multiple times about PrEP. And did he take the advice? I don’t know....But since I’m aware of [mentee’s behavior]...I was like, I’m gonna at least give you [the information]—you can prevent yourself from getting HIV if you take your PrEP every day.

Mentor, age 26 years, Baltimore

When approached by a mentor regarding sexual health in general, mentees reported being generally receptive. For example, if a mentor wanted to casually discuss a mentee’s romantic relationships or healthy relationships, mentees were attentive. However, when these discussions were focused on mentee risk behavior, such as engaging with multiple partners, mentors talked about being less comfortable and sometimes felt embarrassed to bring it up. Mentees in longer mentoring relationships (>1 year) reported that they trusted the mentor to have their best interests in mind when introducing conversations on sexual health, even if it did lead to some embarrassment.

### Mixed Levels of Comfort Mentoring on Sexual Health Topics

When there was sufficient trust in the relationship or observation of potential risk behavior, mentors reported generally feeling comfortable discussing some sexual health topics such as knowing when one is ready to start having sex and using condoms. However, mentors’ stated that confidence in mentoring on other sexual health issues, particularly HIV, varied. Mentors who were living with HIV or who reported being in serodiscordant partnerships communicated higher confidence than those without direct experience:

Besides that, we talked about, you know, [HIV] status...I had wound up finding out not too long before that I was positive, and when I was young, I didn’t have the luxury of having that much insight into the do’s and don’ts of how [HIV] is transmitted. Because of my status, I did my homework and so I know what to say when I talk to [Mentee] about it.

Mentor, age 28 years, Philadelphia

Mentors who reported feeling less comfortable with HIV-related conversations also reported feeling less confident in their knowledge of HIV prevention practices beyond condom use. Conversely, mentors who reported feeling very comfortable and knowledgeable about HIV also reported detailed conversations with their mentee regarding risk reduction strategies through safer sex practices. Mentors who reported identifying as an SGM individual also reported a need to stay updated on information regarding pre-exposure prophylaxis (PrEP); however, in most cases, mentors did not report having detailed conversations about PrEP.

Likewise, mentees did not recall having in-depth conversations with mentors about sexual health and/or HIV unless they had a long-standing, trusting relationship with the adult. For those who did report such discussions, mentees’ reported comfort level appeared to be dependent on multiple factors, including the level of trust, the types of sexual experiences they believed their mentors may have encountered, and whether their mentor has ever implicitly mentioned HIV in previous conversations with the mentee (such as in the context of condom use). When mentees reported being highly trusting of their mentors, they also reported higher comfort levels discussing sensitive health topics with them. In addition, mentees who reported a shared sexual or gender identity with their mentor reported a greater willingness to engage in discussions about sexual health as opposed to a potential mentor without a shared experience:

In the lifestyle I live, people ain’t really there for you. So sometimes you having somebody in your community to mentor you, that can mean a lot because they understand the issues I’m going through. Instead of going to someone that really don’t understand you, I’d rather go to somebody that understands me and accepts me for me.

Mentee, transgender female, age 22 years, Washington, DC

Mentees also reported feeling more comfortable and confident in their mentors’ abilities to provide accurate information about sexual health when mentors had direct experience with the SGM community or identified as SGM individuals.

### Mentees Have Multifaceted Needs and Mobile Health Tools Could Help

This formative research was conducted in large part to learn whether mentors and their mentees would benefit from an app focused on improving mentorship around sexual health issues. Our original conception was that the app would be primarily used by the mentors, with content to be shared with mentees to spark conversations. When asked about their thoughts regarding such a tool, mentors and mentees reported that an app may be useful in facilitating conversations around sexual health topics, particularly as it relates to sharing knowledge, local resources, and communicating about sexual health in a relationship. We did not provide much detail on our conceptualization of such an app because we wanted to learn what would be most useful for the users themselves:

The app sounds like an important thing. Like, someone to remind you that, hey, you have to go to your doctor’s appointment today. Or there’s these new condoms...Did you get these? Have you seen these? There’s a new transgender group going on, I think you should attend, maybe you wanna check it out. So, those things [on the app] would probably make it easy [to use].

Mentor, age 36 years, Washington, DC

We also asked if such an app is something that is needed by the SGM mentoring community, and participants overwhelmingly agreed that it would be highly beneficial to both mentors and mentees.

Most participants felt HIV was an important issue in their community and needs to be addressed, particularly with African American MSM and transgender women. Mentors cited the need for an app to feature a space (such as a forum) where mentors could talk with each other about common HIV-related issues and share information regarding other health conditions and life challenges that the SGM youth face. Mentors pointed out the multifaceted needs their mentees may face, ranging from day-to-day survival assistance to longer term health conditions, and that these factors should be addressed in the app as well. Notably, mental health was highlighted as a critical issue by most mentors:

If you get nothing else from me in this interview, mental health is number one. Mental health, self-esteem, self-worth, depression, anxiety - those are the things that are getting in the way. Those are some of the factors that are leading to this risky behavior.

Mentor, age 38 years, Philadelphia

A potential app’s data security and a visually discreet interface were identified as important components to consider in its development, particularly if the app is focused on HIV and/or SGM youth. Mentors also identified a need for self-training and suggested that the app include skills building resources tailored to the mentors of SGM youth. Responses from mentees regarding what should be incorporated into the app included a way to be linked with resources and information in a discreet way:

I feel like a lot of people wanna do stuff like [test for HIV, learn about PrEP] secretly. So it will be good if we could like, you know, learn about it without everyone knowing.

Mentee, cisgender male, age 21 years, Baltimore

Mentors in each city felt an app focused on improving their knowledge and self-efficacy to mentor on health topics relevant to SGM youth, combined with a place to view successful mentoring on the relevant issues, may be useful in their mentoring practice. Most participants stated that the prospect of an app tailored to the needs of mentors of high-risk youth would be well received in the community.

## Discussion

### Key Findings and Comparison With Previous Research

Participants in our sample tended to form relationships with mentors and mentees in natural ways rather than through formal youth mentoring programs. This is consistent with studies of LGBTQ youths who have mentors, which have shown that such youths are more likely to report having informal mentors than programmatic mentors [27,28]. Natural mentoring relationships in this study were reported to be of longer duration than formal mentoring relationships, which may be a significant advantage for the mentees, as longer lasting relationships have been linked to more positive youth outcomes [41,42]. In addition to LGBTQ youth in general receiving mentorship through informal channels as opposed to formal programs, research has shown that African American youth tend to use natural mentors on a regular basis as well [43], and those with natural mentorships are more likely to experience positive youth outcomes than those who do not [44].

Naturally occurring relationships, as opposed to those tied to a mentoring program, may be especially important to consider when designing programs that use mentors as the intervention vehicle for African American SGM youth, given that such youths are more likely to be reached through informal networks. One study of mature (older) Black MSM reported that most participants agreed that mature Black MSM should mentor younger Black MSM to help them learn from their mistakes and teach them about safer sex [45]. This was seen as a top priority of the mature Black MSM in the study, especially when considering HIV prevention for younger generations. If older generations of African American SGM are eager to impart their knowledge and provide guidance, this may be a key intervention vehicle for both mentoring and health practitioners alike to promote the health and well-being of younger SGM generations. Although evidence regarding the impact of mentoring on health and other outcomes for SGM youth is just starting to emerge [46], this study begins to unpack the mentoring relationship dynamics and characteristics that would make crucial conversations around sexual health and other sensitive issues possible.

Mentors in this study believed that sexual health topics are important in a mentoring relationship, especially when it concerns African American young SGM, and they expressed a desire to improve their capacity to have such conversations. However, mentors generally did not feel prepared to have in-depth discussions beyond sexual debut and condom use, unless they themselves had direct HIV experience. Furthermore, the few mentors from formal programs interviewed did not feel comfortable discussing sexual health topics because such discussions are not in line with the program’s goals. Few mentors felt confident being a strong guide in all things related to sexual health; however, when given the right tools and resources, particularly in the form of a mobile app, the mentors were confident they could grow into such a role.

Participants in this study also expressed a need to focus on the mental health of the mentees. This is consistent with previous research showing that the emotion work involved in navigating a queer identity and maintaining a sense of belonging and security among youth has a stark impact on youth mental health and well-being [47]. Although mental health concerns were not the original focus of the app and this study, this was subsequently added as a module to HIV-focused content based on current findings. In addition, this finding has prompted the research team to make mental health a priority area for future app versions targeting LGBTQ youth populations.

Although research on the mentoring of young African American SGM is still in a nascent stage, the results of this study are consistent with previous findings highlighting the importance of mentorship for this group of youths, particularly to promote health and well-being. A study examining the natural mentoring relationships of young Black MSM showed that participants preferred mentors to be people with whom they shared key attributes and whom they considered successful and resilient in their own lives [48]. The findings of this study echo these previous results, in that mentee participants felt most comfortable with a mentor who shared a lived experience, and mentors who had such a shared experience felt comfortable imparting their knowledge to the young people they mentor.

Previous research on HIV-related apps also highlights areas where mentors can fill crucial gaps for SGM youth at risk. Although there is a proliferation of HIV-related apps, and participants in this study were open to an app focused on mentoring around sexual health issues, the mentors were reluctant to use existing tools because they were not relevant to mentoring relationships. Previous HIV apps have tended to focus on supporting individuals to test or facilitate adherence for those living with HIV [49-51]. Most of these apps also operate with a connection to clinical settings. Although this is important, adolescent and young adult populations are among the least likely demographic groups to seek health care in a clinical setting, considering routine barriers to access, availability, and affordability that they face [52,53]. In addition, few studies have used adult-youth social support networks such as mentoring in developing a mobile health HIV prevention intervention, with available interventions largely focused directly on the individual [54]. No mentors in this sample mentioned using such resources to help guide their mentoring, sometimes even citing stigma around accessing an HIV-related app. Perhaps an app providing sexual health materials for mentors in the context of their mentoring relationship (rather than separately as HIV prevention education) could add a critical element of discretion, potentially benefiting both mentors and mentees in increasing sexual health and reducing risk in this population (SGM of color).

The results of this study also help to throw light on the utility of mentors in addressing sensitive health issues affecting youth, particularly for populations at increased risk. Literature on health outcomes as a result of mentoring is scant and is largely focused on helping youth adapt to chronic illness or disability or looks at substance use prevention (MR Kaufman, unpublished data, 2021, [55,56]). There has not been a comparison of natural mentoring with formal mentoring programs and their respective impact on health outcomes. This would be an important area for future research, especially for marginalized youth at risk of negative health outcomes.

### Limitations

Participants in this study were largely recruited by word of mouth and advertising through mentoring and LGBTQ organizations, establishments, and events, which may have produced a biased sample. We were able to recruit only 1 mentee from a formal program and 6 mentors associated with programs, despite our best efforts to reach out to all known mentoring programs (particularly those serving African American youth) in the 3 cities. In our informal conversations with community members, it became clear that this population (African American SGM) simply does not trust formal mentoring programs. Such youths appear to trust mentors that they come to know through their own communities and social networks. Regardless, the small representation of mentors and mentees in formal mentorships likely had an impact on the results, as the dynamics of such relationships and associated mentoring needs may be different from naturally formed relationships. In addition, given the stigmas regarding sexual and gender minorities and HIV in the African American community [39], participants may have been hesitant to discuss the issues. We attempted to minimize this by employing interviewers whom we hoped would be relatable to the participants.

Eligible mentee participants in this study were older (aged 15-24 years) than the age group for which most youth mentoring literature exists (younger than 18 years). We chose the older age group because our focus was on conversations regarding sexual behaviors. Focusing on younger mentees may not have revealed the presence of sexual health conversations because they were not yet relevant for younger youth.

Although we included transgender female participants, we did not have a large enough subgroup sample size to make meaningful comparisons with the cisgender male participants. Furthermore, all of the transgender participants were referred to the study by a social support organization that focuses on addressing the basic needs of youth. Interviews with transgender mentees and their mentors tended to focus on mentoring regarding immediate basic needs such as food security, employment, and housing, as these are the services provided by that organization. Future research should further investigate the broader needs of transgender youths who have mentors using a larger and more representative sample of these young persons.

### Conclusions

For the African American SGM community, access to natural mentors may be crucial for young people at risk of HIV to learn to develop healthy behaviors and relationships. Such youth also require mentorship on other health areas (particularly mental health) and life skills. This study highlights the role of mentors for such youth and what is needed for mentors to confidently and effectively address sexual health and other issues with these young people. A resource for mentors to confidently have discussions on critical but sensitive issues with mentees appears promising as a way to promote healthy practices in this vulnerable adolescent population. A mobile app that is discrete and up to date and provides support and resources for the mentors holds promise for meeting some of these needs. Future research on the mentorship of SGM youth of color may also benefit from focusing on naturally occurring mentorship in addition to formal mentoring programs. A mobile app resource for mentors must take into account the fact that many African American SGM seek mentors through informal networks. Through a better understanding of the needs of this particular youth group and their areas of comfort and discomfort when it comes to being mentored by trusted adults, mentoring programs and health practitioners will be better able to address their needs and promote youth health and well-being.

## Appendix

Multimedia Appendix 1 Mentor interview guide summary.

## Abbreviations

LGBTQ: lesbian, gay, bisexual, transgender, and queer
MSM: men who have sex with men
NIH: National Institutes of Health
PrEP: pre-exposure prophylaxis
SGM: sexual and gender minority
