# Supplementary material for: Mentoring Young African American Men and Transgender Women Who Have Sex With Men on Sexual Health: Formative Research for an HIV Mobile Health Intervention for Mentors
Source: JMIR Form Res. 2020 Dec 17;4(12):e17317. doi: 10.2196/17317 (PMC7775199; doi:10.2196/17317)
Supplement: Multimedia Appendix 1 [file formative_v4i12e17317_app1.docx]

**Supplementary File 1.**  Mentor Interview Guide Summary^*^

| **Interview Topic Area** | **Key Questions** |
| --- | --- |
| Background & Mentoring Relationship Origin | - *What does “mentoring” or having a mentee mean to you?* - *How long have you been a mentor to [mentee]?* - *How often do you and your mentee see each other? What do you do together?* - *How often do you communicate with your mentee when you do not see each other (in person)? What do you use to communicate?* - *What topics do you mentor on most often?* - *What are you hoping to accomplish by being a mentor to [mentee]?* - *What has been easy/fun in mentoring [mentee]? What has been challenging/difficult?* |
| Mentoring on Sex and HIV | - *Do you ever discuss sexuality, sexual identity, gender identity, or sexual behavior with [mentee]?* - *Can you please tell me if you have talked about any of the following with your mentee?*    - *Types of sexual activity*   - *Types of sexual partners*   - *HIV risk behavior*   - *PrEP*   - *HIV testing*   - *HIV treatment*   - *STIs* - *How comfortable are you talking about these sex-related topics with [mentee]?* |
| Potential Utility of App for HIV-related Mentoring | - *What barriers do you think you would encounter if you tried to discuss some of these topics with [mentee]?* - *What would make it easier for you to mentor [mentee] on sex-related topics?* - *If there were an app that could help, what would it look like?* - *Do you think you would use such an app with [mentee]?* |

^*^Mentee interview guide was similar, but questions were asked from their perspective.
